# Enhancing the research and publication efforts of health sciences librarians via an academic writing retreat

**DOI:** 10.5195/jmla.2017.320

**Published:** 2017-10-01

**Authors:** John W. Bullion, Stewart M. Brower

## Abstract

**Background:**

This case study describes the South Central Chapter of the Medical Library Association (SCC/MLA) initiative to develop an academic writing retreat for members who sought the necessary time and support to advance their research projects toward publication.

**Case Presentation:**

SCC/MLA staged a dedicated writing retreat to coincide with the organization’s 2012, 2013, and 2014 annual meetings. Each cohort met over two days to write and to workshop their peers’ manuscripts. Organizers distributed an online survey one month after each retreat to evaluate attendees’ perceptions.

**Conclusions:**

Three years’ worth of writing retreats yielded fourteen peer-reviewed articles and one book chapter. Participants indicated that the retreat helped them meet or exceed their writing goals by offering protected time and a setting conducive to productivity. The format of the retreat is cost effective and easily adaptable for fellow professionals who wish to organize a formal event as a conference offering or simply support a writing group at their home institutions. In SCC/MLA, the retreat revitalized interest in writing and demystified the scholarly publication process.

## BACKGROUND

Writing for publication can be intimidating and frustrating, and the need for time and support to write is common across a wide variety of academic disciplines [1, 2]. Many scholars have championed the concept of an academic writing retreat as an opportunity to write in a distraction-free environment and receive feedback from peers. In their recent integrative review, Kornhaber et al. identified five main benefits of a writing retreat: protected time and space, development of academic writing competence, creation of a community of practice, organizational investment in the form of experienced mentors and follow-up opportunities, and intrapersonal benefits, such as increased motivation and reduced writing-related anxiety [3].

Retreat facilitators have developed many variations on the model. Tysick and Babb’s 2006 study, the only appearance of the concept in the library literature, described an academic writing group at the University of Buffalo that offered graduate library students expert feedback from experienced facilitators, support from colleagues, and ample time to write [4]. While the library students in the Tysick and Babb study convened on campus, participants in residential retreats such as those described by Grant enjoyed “marvellous food...and proximity to hot pools for evening soaking” in a pastoral setting meant to spur productivity [5]. Antoniou and Moriarty balanced writing to the precise specifications of scholarly publication with “left-brain” writing exercises geared toward sparking participants’ creativity [6]. Others such as Silvia [7] and Murray and Newton [8] developed a more process-oriented model, requiring reasonable but concrete writing goals and maintaining accountability by regularly monitoring writing progress. Rankin’s inclusive, multidisciplinary group model accommodated work at all stages of the writing process, from initial drafts to “near-final” manuscripts, requiring each participant to pose targeted questions to ensure useful feedback [9].

The retreat concept even appears frequently in clinical literature as a means of offering dedicated writing time to medical and nursing faculty with heavy teaching and clinical workloads [10–15]. While health sciences librarians offer these dual-role caregivers invaluable research assistance in the form of literature searching, document delivery, and citation management, when it comes to their own scholarly output, many librarians never pursue their own research opportunities beyond papers and posters presented at professional meetings [16]. In the end, health sciences librarians find themselves stymied by lack of time, geographic isolation from coauthors and peers, or trepidation about writing and publication [17–19].

## STUDY PURPOSE

In an attempt to bolster the chapter’s scholarly activity, the South Central Chapter of the Medical Library Association (SCC/MLA) staged an “academic writing retreat” during three consecutive years at the organization’s annual meeting to provide members with protected time to write and a peer-support network to help advance research projects toward publication. By the end of each two-day retreat, participants were expected to demonstrate significant progress on their manuscripts, apply revision suggestions from peers and facilitators, determine the optimal journals to submit their manuscripts to, and develop a concrete plan for their writing after the retreat.

## CASE PRESENTATION

Because SCC/MLA covers a five-state area (Arkansas, Louisiana, Oklahoma, New Mexico, and Texas), staging a residential retreat in a “pastoral” location proved logistically and financially impossible. With an eye toward openings in members’ already busy calendars and with the financial blessing of the South Central Academic Medical Libraries Consortium (SCAMeL), organizers opted to schedule the retreat concurrently with SCC/MLA’s annual conference so that the cost of registration and lodging would already be borne by attendees’ home institutions. At each conference, the nearest academic medical library agreed to host the retreat free of charge. The only costs incurred by SCAMeL were the catered meals (breakfast and lunch) for attendees and printing fees for the informational packet and participants’ manuscripts. Transportation to and from the retreat site was largely handled by the attendees themselves the first year, although with the help of local arrangements committees, facilitators were able to arrange complimentary shuttle service to and from conference hotels for the second and third retreats. The total cost of the retreat never exceeded $1,000.

Each retreat coincided with SCC/MLA’s annual meetings in Lubbock, Texas, in 2012, and Fort Worth, Texas, in 2013, as well the 5-chapter “Quint” meeting in Denver, Colorado, in 2014. Organizers opened the “Quint” retreat to outside applicants, with priority given to SCC/MLA members. In the chapter newsletter and email discussion list, organizers asked applicants to submit a paragraph-length summary of their topics and writing goals. Organizers encouraged members to apply whether they had a completed draft in hand or only a rough outline and welcomed all levels of experience, from novice scholars to experienced researchers. All submissions from 2012–2014 met these minimum requirements, resulting in a 100% acceptance rate. The retreat maintained a consistent level of participation, starting with a cohort of 9 attendees in 2012, 11 in 2013, and 10 in 2014. The 2013 and 2014 retreats allowed prior participants to reapply, provided that their submissions indicated either distinct progress or new writing goals. There were 5 return attendees (3 in 2013 and 2 in 2014). No SCC/MLA member participated in all 3 retreats.

On day one, facilitators distributed information packets containing each participant’s project summaries and writing goals, as well as explanations of the workshop method, writing exercises, and instructions to authors for several professional journals, including the *Journal of the Medical Library Association* and *College & Research Libraries* (supplemental Appendix A). Facilitators encouraged outside attendees at the “Quint” retreat to use the packet as a template to administer the same workshop model in their chapters or at their home institutions.

Daily lunches and refreshments were provided. Facilitators also built “decompression time” (full group meetings) into the day’s activities to provide a forum to discuss progress. At the end of the first day, participants distributed hard copies of their drafts to the group. To maximize workshop time, facilitators required manuscripts to include at least three targeted questions. Attendees were asked to make these questions as specific as possible (e.g., “Is the methodology section detailed enough?” and “Is the literature review thorough?”) to help readers assess whether the writer’s stated goals were met and to suggest further steps. This format allowed group members with varying levels of experience to quickly identify and respond to perceived problem areas in their peers’ manuscripts, thus ensuring that each writer would have their main concerns addressed.

The following morning, each manuscript was allotted between twenty and thirty minutes for a facilitator-guided workshop discussion among the full group, who addressed the writer’s questions before embarking on a broader exploration of other approaches that the writer might consider moving forward. After the workshop concluded, participants were granted six more hours of writing time to incorporate suggested revisions. At the end of day two, the entire group reconvened to debrief and set goals beyond the retreat.

**Figure 1:**
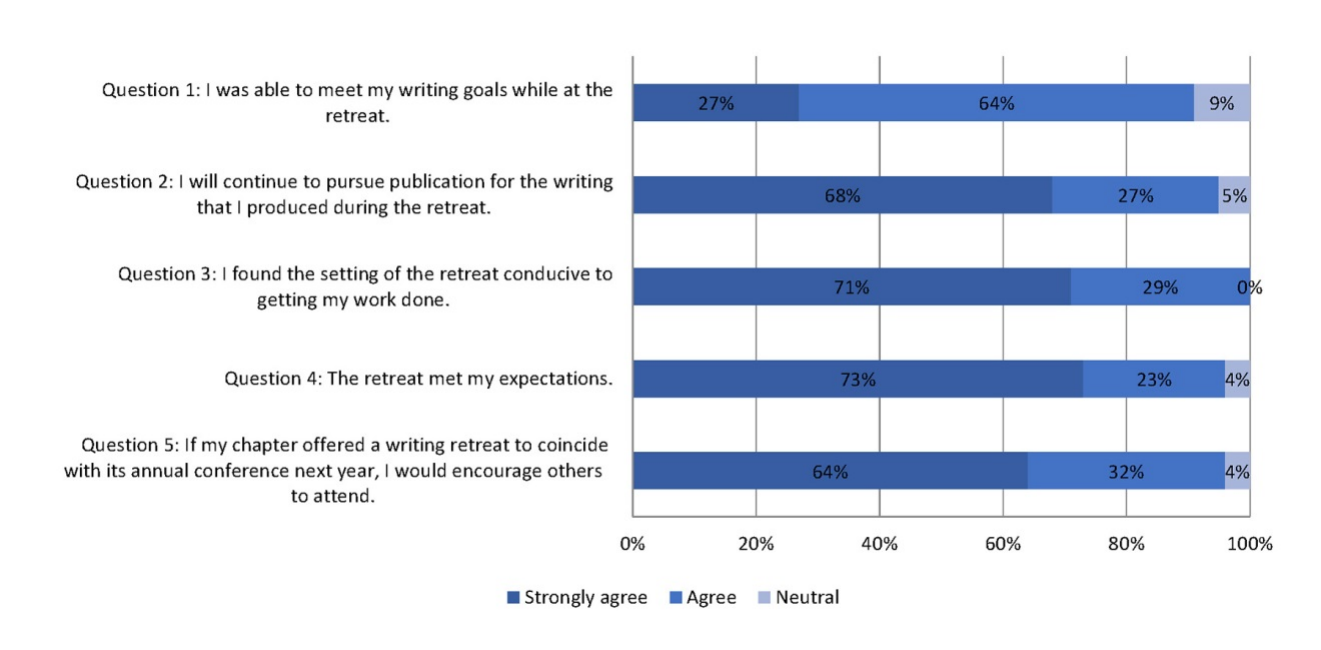
Survey responses from post-retreat evaluation Attendees were asked to evaluate each statement using a scale of 1 (strongly disagree) to 5 (strongly agree).

Organizers distributed an online survey via SurveyMonkey one month after each retreat. The survey consisted of 5 statements, rated on a 5-point Likert scale (supplemental Appendix B). A final open-ended question offered space for detailed comments or suggestions. Organizers did not require respondents to submit identifying information such as name, home institution, educational level, or prior publication experience. Of the 30 total participants at the 3 annual writing retreats, 22 completed the survey for an overall response rate of 73%. Figure 1 shows a chart mapping post-retreat survey evaluations from 2012–2014. Respondents rated their experience highly, with “Strongly agree” ratings averaging 69% across questions 2–5; the most negative rating on any of the closed questions was “Neutral.” Question 1 received a slightly higher percentage of “Neutral” ratings (9%) and a significantly lower percentage of “Strongly agree” ratings (27%). Many attendees reported that peer feedback had compelled them to reconsider their initial writing goals, which might account for the relatively lower percentages on Question 1.

While the survey provided organizers with a useful record of positive in-the-moment perceptions, the true measure of the retreat’s impact on scholarly activity depended largely on participants continuing to push toward publication. Over time, retreat participants made many contributions to the published literature. Three years’ worth of writing retreats yielded fourteen peer-reviewed articles and one book chapter. The citations listed in Appendix C reflect the publication records of 2012–2014 retreat attendees (supplemental Appendix C).

## DISCUSSION

Based on survey responses, medical libraries provided an ideal retreat setting, and staging the event during the annual conference eliminated the obstacle of geography for a time. One respondent noted that “everything about the [medical library] space led to a congenial & productive atmosphere,” while another maintained that they were “able to focus more so than i [*sic*] would if i [*sic*] did not attend the retreat.” However, the limits of this approach became obvious post-retreat, as participants returned to their home institutions and writing became a less immediate priority. One repeat attendee noted, “I wouldn’t have minded an extra push [from peers and facilitators] a few months after the retreat to see if I was keeping on the writing track.” Another suggested “setting [additional] goals post-retreat” to increase accountability.

Lingering questions about lack of follow-up (not to mention the difficulty of traveling outside the region) might have also contributed to lower member attendance at the 2014 “Quint” retreat in Denver, whose cohort only included one new and two returning SCC/MLA applicants. In recognition of these concerns, the “Quint” retreat was the first to employ a shared Google Drive, where attendees could upload revised manuscripts and follow up with feedback post-retreat.

Even though each retreat coincided with the chapter’s annual meeting, certain professionals still faced barriers to participation. Twenty-nine of the thirty total attendees over the 2012–2014 period were affiliated with an academic medical library. No hospital librarians from SCC/MLA ever attended; the lone hospital librarian who attended any of the three retreats was from outside the chapter. In previous studies on the research habits of health sciences librarians, hospital librarians overwhelmingly cited lack of time and institutional support as obstacles to participation in scholarly activity [20, 21]. These same barriers, it appears, are shared by hospital librarians in SCC/MLA, many of whom face difficulty simply attending an annual meeting, much less participating in a concurrently held retreat.

The publication tally, while robust, highlighted the main limitation of the study: the organizers’ failure to offer a basis of comparison by accurately calculating the publication rate of SCC/MLA members prior to the 2012 retreat. However, it remains unclear how organizers would have successfully argued that any quantifiable uptick in scholarly activity could be directly attributed to the retreat. A list of publications by retreat attendees may offer some evidence of impact, but much like the enthusiastic survey responses, it fails to answer key questions about the motivations of participants, such as without the intervention of a retreat, might some novices have grown discouraged and never published? Would more experienced participants have published anyway? More broadly, are nonparticipants with extensive publication records already involved in informal writing groups at their home institutions or among their colleagues? These questions serve as a source of further inquiry to determine the true impact of a writing retreat on the scholarly activity of health sciences librarians.

## CONCLUSIONS

The SCC/MLA writing retreat offered chapter members protected time to write and a crucial peer-support network to gain feedback on their manuscripts. Survey responses indicated an obvious interest in staging future retreats, and organizers hope to employ online workspaces to track attendees’ post-retreat progress, as well as to emphasize elevating professionals who face increased barriers to writing, such as hospital librarians. The format of the retreat proved cost effective and readily adaptable as a conference offering or as a template for more informal writing groups (in-person or online). Most importantly, the retreat revitalized interest in writing, demystified the scholarly publication process, and enabled members of SCC/MLA to contribute to a dynamic professional knowledgebase.

## SUPPLEMENTAL FILES

Appendix A South Central Chapter of the Medical Library Association Academic Writing Retreat information packets

Appendix B Survey instrument

Appendix C List of publications from the members of the South Central Chapter of the Medical Library Association Academic Writing Retreat
