# Supplementary material for: Enhancing the research and publication efforts of health sciences librarians via an academic writing retreat
Source: J Med Libr Assoc. 2017 Oct 1;105(4):394–9. doi: 10.5195/jmla.2017.320 (PMC5624429; doi:10.5195/jmla.2017.320)
Supplement: Appendix A [file jmla-105-394-s001.pdf]

JMLA Journal of the Medical Library Association

SC

Supplemental content to
J Med Libr Assoc. Oct;105(4): dx.doi.org/10.5195/jmla.2017.320
www.jmla.mlanet.org
© Bullion, Brower 2017

# Enhancing the research and publication efforts of health sciences librarians via an academic writing retreat

**John W. Bullion, MFA, MSLS, AHIP; Stewart M. Brower, MLIS, AHIP**

## APPENDIX A

### South Central Chapter of the Medical Library Association Academic Writing Retreat information packets

### Agenda

**Friday, October 2**

| | |
|---|---|
| 9:00 a.m.–9:30 a.m. | Introductions & Opening Remarks |
| 9:30 a.m.–11:30 a.m. | ***Writing Time*** |
| 11:30 p.m.–noon | **Decompression** |
| noon–1:00 p.m. | Lunch |
| 1:30 p.m.– 4:30 p.m. | ***Writing Time*** |
| 4:30 p.m.–5:00 p.m. | **Decompression** |

At the end of Friday, participants will be asked to upload a copy of their manuscript to share with the group. Saturday morning's workshop session will be devoted to reviewing these manuscripts with one another before we all get back to the writing.

**Saturday, October 3**

| | |
|---|---|
| 9:00 a.m.–10:00 a.m. | ***Writing Time*** |
| 10:00 a.m.–noon | Review; **Workshopping** |
| noon–1:00 p.m. | Lunch |
| 1:00 p.m.–:30 p.m. | Group Photo |
| 1:30 p.m.–4:30 p.m. | ***Writing Time*** |
| 4:30 p.m.–5:00 p.m. | **Final Decompression** |

Some definitions:

- Writing Time: Full group quiet time with focus on individual writing, although facilitators will be available around the clock to review manuscripts.
- Decompression: Full group discussion with emphasis on checking in with each other, supporting each other's writing goals.
- Workshopping: Full group discussion with emphasis on writing technique, constructive critiquing of our work.

**Acceptance Rates**

For some of the journals being considered for submission, here are their acceptance rates for publication:
*College & Research Libraries:* 30% to 40%
*Journal of Academic Librarianship:* 40% to 45%
*Journal of the Medical Library Association (JMLA):* 45%
*LIBRES:* 21% to 30%
*Medical Reference Services Quarterly:* 40% to 50%
*Science & Technology Libraries:* 40% to 50%

JMLA Journal of the Medical Library Association

SC
Supplemental content to
J Med Libr Assoc. Oct;105(4): dx.doi.org/10.5195/jmla.2017.320
www.jmla.mlanet.org
© Bullion, Brower 2017

**Participants and proposals**

**Attendee**
**Louisiana State University**
"I would like to use the writing retreat to work on an article for a *Reference Services Review* theme issue on health literacy and libraries. My article is a collaboration; it will explore ways librarians can help increase health literacy within the d/Deaf community. This writing project will describe the challenges faced by the d/Deaf community as well as the diversity within this community. During the writing retreat I would like to focus on synthesizing what I have learned about this population and the ideas I have on improving health literacy. My goal is to write about 2,000 words."

**Attendee**
**MD Anderson Cancer Center**
"I am working on an EdD in Education Technology and my research topic is online patient education. Specifically, I am interested in doing a mixed methods study measuring patient engagement after a didactic online orientation to a cancer center compared to a patient directed online orientation. I need to work on my literature review and my research proposal but for this retreat I am planning on focusing on the literature review."

**Attendee**
**University of Arkansas for Medical Sciences Library**
"This is my writing retreat. I have been examining various citation managers and reasons why libraries choose specific citation managers and how they are used. I conducted a survey, which just closed today. My goals for the retreat are to pull together the literature review and survey results and write a paper for *JMLA."*

**Attendee**
**Louisiana State University Health Shreveport**
"After a contributed paper at SCC (2013), paper at LLA (2014), and poster at MLA (2015), I need to actually start publishing articles about the 2013 NN/LM SCR Disaster Preparedness Award titled *Are You Prepared? Promoting Disaster Preparedness in Northwest Louisiana through the Public Libraries*. I would like to initially focus on the work with the public library systems, emphasizing the cooperation in proposing and conducting the award project and detailing the different programs and what the staff did at the branches and at other activities, such as the Red River Revel. My goal is to get a first draft from the two days of writing aimed for either *Public Libraries* or *Louisiana Libraries*. Later I want to write a second article focused on the medical librarians' roles and outreach for publication in *JMLA* or another library journal oriented to medical librarianship and/or community disaster preparedness of these publications."

**Attendee**
**MD Anderson Cancer Center**
"I am interested in ways to market our online resources and have been curious about the experience of our users when they access our subscription resources. [Name redacted] and I are hosting a round table at SCC to share our initial findings. During my time at the workshop I plan to summarize background information from my literature review and our findings. I also want to explore the elements of a white paper for reporting versus a descriptive study."

SC
Supplemental content to
J Med Libr Assoc. Oct;105(4): dx.doi.org/10.5195/jmla.2017.320
www.jmla.mlanet.org
© Bullion, Brower 2017

**Attendee**
**Collin College**
"This is my first time at the retreat. My goal is to expand my MLA paper presentation into an article for *MLA News* on the NLM Community College Outreach project and its collaboration with Collin College. My other goal is to write a review of BoardVitals from STAT!Ref."

**Attendee**
**University of North Texas**
"My purpose in attending the writing workshop is to gain a safe place to write and work alongside another attendee on our project writing about health literacy and the Deaf community. I hope to learn and create while gathering all the information we have on our topic and make a whole that can help people better understand the underserved community of the Deaf. My goal is collaboration with [name redacted] and a substantial chunk of the paper written. As she already posted, about 2000 words is the goal."

**Attendee**
**University of Oklahoma Health Sciences Center**
"This is my first time at the retreat and I plan to work on writing my dissertation proposal. My main focus is the literature review. I have done all the research and just need to review it and write it up. While the focus will be on the literature review my secondary goal is to have an initial draft of the proposal finished by the end of the retreat. My dissertation is looking at the use of decision support systems (such as dynamed and isabel) in critical care units and whether or not they have an impact on diagnosis."

**Attendee**
**Texas A&M University**
"I'm building a template for the Medical Sciences Library's annual report and will begin writing the features in the report, which will likely include a brief history of the library."

## Workshopping method

Workshopping is a method for leading a constructive discussion of any participant's writing in progress. During the retreat, we will have specific times where we will workshop other participants' manuscripts to provide them with useable feedback to assist them with their writing. To have the maximum value, the following process will be followed:

### 1. *Questions and goals*

The author will begin by leading off with two or three questions that he or she would like to have answered. These questions usually take the form of something directed at the structure or comprehensibility of the writing but can be directed at their own writing goals as well. Some examples of these kinds of questions might be:

- "Did you follow the transition between the second section and the third?"
- "There are three authors. Do we have a consistent, neutral voice?"
- "Do I do enough to stress the importance of planning for this kind of outreach?"
- "Is the tone appropriate for this kind of article?"

Journal of the Medical Library Association
Supplemental content to
J Med Libr Assoc. Oct;105(4): dx.doi.org/10.5195/jmla.2017.320
www.jmla.mlanet.org
© Bullion, Brower 2017

### 2. *Positive feedback*

All the other participants will then take turns saying something positive about the manuscript. No one has to say anything directed at the author's questions, but all remarks for the first round of discussion should be of a strong, positive nature.

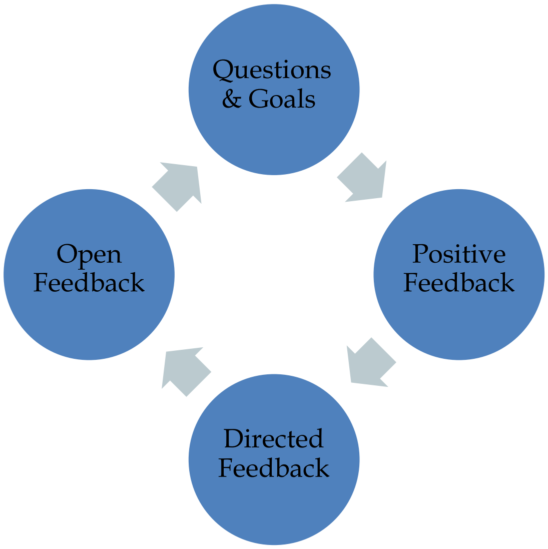


### 3. *Directed feedback*

The second round of discussion will be focused on the questions that were asked by the author. Each participant should make an effort to respond directly to the questions that were asked and not elaborate on other matters related to the manuscript. This feedback should be honest and open. In most cases, it is best to respond to the questions only with answers, not with recommendations on best methods to make corrections. For example, a participant might say about a multi-authored work, "I did not feel that you have achieved a neutral voice. I could tell right away that the results section was written by a different person than the methodology section. Many words and phrases were inconsistently used." Note that in this example, the participant is not recommending changes, only pointing out what they read, consistent with the questions asked.

### 4. *Open feedback*

The final round of discussion is not directed at any particular question or goal, but rather is meant to address any other matters the participants want to bring up. Gentle suggestions about what might be done to correct features of the writing may be addressed, but more direct instruction (i.e. "What you really should do is…") should be avoided.

Remember that the overall goal of workshopping is to provide positive, constructive feedback that the author can consider and use to improve their writing. Therefore, all commentary should be directed at the writing itself and not at the author.

JMLA Journal of the Medical Library Association

SC

Supplemental content to
J Med Libr Assoc. Oct;105(4): dx.doi.org/10.5195/jmla.2017.320
www.jmla.mlanet.org
© Bullion, Brower 2017

**Independent writing exercises**

Sometimes, the writing does not come easily. During the retreat, if you find yourself facing a bit of a block, you may want to try your hand at one of the following writing exercises. These are entirely optional and are each designed to give you a new perspective on your manuscript.

*Focus exercise:* Write a single sentence that describes the major point of your manuscript. Use the following format. "What it all boils down to is: _________."

*Flash essay:* In 10 minutes, write no more than 500 words that summarize one aspect of your larger work. For example, select one section of your manuscript and write a flash essay that summarizes just that section.

*The email:* Imagine that all the work is done, and you've gotten published. To make things even better, a librarian has written to you out of the blue, complimenting your work and identifying what they liked best about your article. Write a 250 word email reply to this person. What do you think was their favorite part?

*Three new things:* List three things that you now know that you didn't know when you began your research for your manuscript.

**Thoughts on writing**

"Writers may be solitary but they also tend to flock together: they like being solitary together."

Neil Gaiman

"Planning to write is not writing. Outlining, researching, talking to people about what you're doing, none of that is writing. Writing is writing. "

E.L. Doctorow

"I think most writers, even the best, overwrite. I prefer to underwrite. Simple, clear as a country creek."

Truman Capote

"Either write something worth reading or do something worth writing."

Benjamin Franklin

"If you can do a half-assed job of anything, you're a one-eyed man in the kingdom of the blind."

Kurt Vonnegut, from *Player Piano* (1952)

"There is nothing to writing. All you do is sit down at a typewriter and bleed."

Ernest Hemingway

"When struggling to write their first article, some writers lament, "Why would they care about my research?" If *they* refers to the world at large, I can assure you that they are uninterested in your research. But if *they* refers to researchers in your area, then you should expect some interest in your article…To write good articles, master the article formula, submit pristine first drafts, and craft excellent resubmission letters. You'll find that the world of journals isn't scary: It's merely slow."

Paul J. Silva PhD, from *How to Write a Lot* (2007)

JMLA Journal of the Medical Library Association

SC

Supplemental content to
J Med Libr Assoc. Oct;105(4): dx.doi.org/10.5195/jmla.2017.320
www.jmla.mlanet.org
© Bullion, Brower 2017

"A pen is certainly an excellent instrument to fix a man's attention and to inflame his ambition."

John Adams

"The more I think about our species the more I think we just do stuff and make up explanations later when asked. But it's not true that I would rather write than read. I would rather read than write. To be honest I would rather hang upside down in a bucket than write."

Douglas Adams

"In this book, I argue that elegant ideas deserve elegant expression; that intellectual creativity thrives best in an atmosphere of experimentation rather than conformity; and that, even within the constraints of disciplinary norms, most academics enjoy a far wider range of stylistic choices than they realize."

Helen Sword, from *Stylish Academic Writing*

"We all often feel like we are pulling teeth, even those writers whose prose ends up being the most natural and fluid."
"Almost all good writing begins with terrible first efforts."
"Perfectionism is the voice of the oppressor, the enemy of the people. It will keep you cramped and insane your whole life and it is the main obstacle between you and a shitty first draft."
"I wish I felt that kind of inspiration more often. I almost never do. All I know is that if I sit there long enough, something will happen."

Four quotes by Annie Lamott, from *Bird By Bird*

"The difference between the almost right word and the right word is…the difference between the lightning bug and the lightning."

Mark Twain

"Vigorous writing is concise. A sentence should contain no unnecessary words, a paragraph no unnecessary sentences, for the same reason that a drawing should have no unnecessary lines and a machine no unnecessary parts. This requires not that the writer make all his sentences short, or that he avoid all detail and treat his subjects only in outline, but that every word tell."

William Strunk, Jr. and E.B. White, from *The Elements of Style*

"Save words."

"Kill your darlings."
