# Supplementary material for: Enhancing the research and publication efforts of health sciences librarians via an academic writing retreat
Source: J Med Libr Assoc. 2017 Oct 1;105(4):394–9. doi: 10.5195/jmla.2017.320 (PMC5624429; doi:10.5195/jmla.2017.320)
Supplement: Appendix B [file jmla-105-394-s002.pdf]

JMLA Journal of the Medical Library Association

SC
Supplemental content to
J Med Libr Assoc. Oct;105(4): dx.doi.org/10.5195/jmla.2017.320
www.jmla.mlanet.org
© Bullion, Brower 2017

Enhancing the research and publication efforts of health sciences librarians via an academic writing retreat

John W. Bullion, MFA, MSLS, AHIP; Stewart M. Brower, MLIS, AHIP

APPENDIX B

Survey instrument

South Central Chapter of the Medical Library Association (SCC/MLA) writing retreat survey questions

1. I was able to meet my writing goals at the retreat.
a. Strongly disagree
b. Disagree
c. Neutral
d. Agree
e. Strongly agree

2. I will continue to pursue publication for the writing that I produced during the retreat.
a. Strongly disagree
b. Disagree
c. Neutral
d. Agree
e. Strongly agree

3. I found the setting of the retreat conducive to getting my work done.
a. Strongly disagree
b. Disagree
c. Neutral
d. Agree
e. Strongly agree

4. The retreat met my expectations.
a. Strongly disagree
b. Disagree
c. Neutral
d. Agree
e. Strongly agree

5. If organizers offered a writing retreat to coincide with the annual SCC/MLA conference next year, I would encourage others to attend.
a. Strongly disagree
b. Disagree
c. Neutral
d. Agree
e. Strongly agree

Other comments/suggestions for improvement?
