# Supplementary material for: Enhancing the research and publication efforts of health sciences librarians via an academic writing retreat
Source: J Med Libr Assoc. 2017 Oct 1;105(4):394–9. doi: 10.5195/jmla.2017.320 (PMC5624429; doi:10.5195/jmla.2017.320)
Supplement: Appendix C [file jmla-105-394-s003.pdf]

Journal of the Medical Library Association

Supplemental content to
J Med Libr Assoc. Oct;105(4): dx.doi.org/10.5195/jmla.2017.320
www.jmla.mlanet.org
© Bullion, Brower 2017

Enhancing the research and publication efforts of health sciences librarians via an academic writing retreat

John W. Bullion, MFA, MSLS, AHIP; Stewart M. Brower, MLIS, AHIP

APPENDIX C

List of publications from the members of the South Central Chapter of the Medical Library Association Academic Writing Retreat

Goodman X. The ALA book of library grant money, by Nancy K. Maxwell [book review]. Med Ref Serv Q. 2016 Jan–Mar;35(1):130–2.

Godbey S, Wainscott S, Fawley N, Goodman X. Ethnography in action: active learning in academic library outreach to middle school students. J Libr Admin. 2015 Jul;55(5):362–75.

Hoberecht T, Randall K, Schweikhard AJ. Library tutorials in an allied health evidence-based practice class. Med Ref Serv Q. 2015 Apr–Jun;34(2):240–8.

Huslig MA, Vardell E. ClinicalKey 2.0: upgrades in a point-of-care search engine. Med Ref Serv Q. 2015 Jul–Sep;34(3):343–52.

Knapp M. Instruction in health sciences libraries. In: Wood MS, ed. Health sciences librarianship. Lanham, MD: Rowman & Littlefield; 2014. p. 275–98.

Knapp M. Technology for one-shot instruction and beyond. J Electron Resour Med Libr. 2014 Oct;11(4):217–25.

Knapp M, Brower S. The ACRL framework for information literacy in higher education: implications for health sciences librarianship. Med Ref Serv Q. 2014 Oct–Dec;33(4):460–8.

Peters C, Vaughn P. Initiating data management instruction to graduate students at the University of Houston using the New England Collaborative Data Management Curriculum. J eScience Librariansh. 2014 Dec;3(1):86–99.

Phillips DY, Walsh B, Bullion JW, Reid PV, Bacon K, Okoro N. The intersection of intimate partner violence and HIV in US women: a review. J Assoc Nurs AIDS Care. 2014 Jan–Feb;25(1 suppl): S36–S49.

Rey LM, Croft VF, Whitaker, SK, Stephens G. Impacting librarianship and veterinary medicine: history of the Veterinary Medical Libraries Section of the Medical Library Association from 1974 to 2014. J Agric Food Inf. 2015 Jul;16(3):253–70.

Schweikhard AJ. An information needs assessment of school nurses in a metropolitan county. Med Ref Serv Q. 2016 Jan–Mar;35(1):27–41.

Shurtz S, Sewell R, Halling TD, McKay B, Pepper C. Assessment of an iPad loan program in an academic medical library: a case study. Med Ref Serv Q. 2015 Jul–Sep;34(3):265–81.

Vaughn P. Analysis for science librarians of the 2014 Nobel Prize in chemistry: the work of Stefan Hell, William E. Moerner, and Eric Betzig. Sci Technol Libr. 2015 Jan–Mar;34(1):32–42.

Vassar M, Carr B, Kash-Holley M, DeWitt E, Koller C, Day J, Day K, Herrmann D, Holzmann M. Database choices in endocrine systematic reviews. J Med Libr Assoc. 2015 Oct;103(4):189–92. DOI: http://dx.doi.org/10.3163/1536-5050.103.4.005.

Wilson G. The process of becoming an embedded curriculum librarian in multiple health sciences programs. Med Ref Serv Q. 2015 Oct–Dec;34(4):490–7.
